# Dynamic Mechanical Properties and Constitutive Modeling of Polyurethane Microporous Elastomers

**DOI:** 10.3390/polym16213056

**Published:** 2024-10-30

**Authors:** Huiming Liu, Youcai Xiao, Yu Zou, Yong Han, Chenyang Fan, Yi Sun

**Affiliations:** 1 College of Mechatronic Engineering, North University of China, Taiyuan 030051, China; 15536877107@139.com; 2 No. 601 Institute of the Sixth Academy of CASIC, Hohhot 010076, China; 3 Science and Technology on Electromechanical Dynamic Control Laboratory, Xi’an 710000, China; 4 Departments of Astronautic Science and Mechanics, Harbin Institute of Technology, Harbin 150001, China; sunyi_hitgroup@163.com

**Keywords:** PUEs, mechanical properties, strain rate effect, density effect, constitutive model

## Abstract

The present study fabricated samples of polyurethane elastomers (PUEs) with three distinct densities and assessed their mechanical responses using split Hopkinson pressure bar (SHPB) tests. The findings reveal a significant increase in PUE stress with increasing strain rate and density. To further investigate the influence of strain rate sensitivity on PUEs, a strain rate sensitivity coefficient was employed to quantify the impact of strain rate on the mechanical properties of PUEs. Separate quantifications were performed for collapse stress, plateau stress, and densification strain as indicators of the strain rate sensitivity coefficient. The results demonstrate that the collapse stress sensitivity coefficient was notably affected by the applied strain rate. Additionally, both collapse and plateau stresses exhibited an increase with increasing density, which could be described by a power function relationship. Based on the theory of strain energy function, a constitutive model considering density and strain rate effects was developed to describe the stress–strain behavior of PUEs under various densities and strain rates. A comparison between this constitutive relationship and experimental results showed good agreement, highlighting its potential in describing dynamic mechanical behavior.

## 1. Introduction

The mechanical properties of polyurethane elastomers (PUEs) and similar elastic materials encompass a wide range, rendering them highly suitable for protective structures like sandwich panels and impact-resistant helmets in armor applications [1,2,3,4]. The design objective of these structures is to mitigate severe injuries caused by high-velocity impacts, leveraging the unique attributes of PUEs, including its exceptional resilience, energy absorption capacity, and vibration damping capabilities. PUEs, as a class of advanced polymers, display a complex set of dynamic responses. These include significant nonlinear deformations under extensive elongations [5] and a notable sensitivity to strain rates that varies with different rates [6,7]. The comprehensive understanding of PUEs’ mechanical attributes is essential for the development and assessment of protective capabilities in structures designed to withstand impacts. Furthermore, such insights are indispensable for determining the material properties required for precise computational modeling of structural dynamics.

In the past decade, numerous scholars have dedicated themselves to investigating the mechanical properties of PUEs across a wide range of strain rates, emphasizing the modifications in behavior observed under both quasi-static and dynamic conditions [8,9,10,11,12,13]. The work of Yi et al. [14] is particularly noteworthy for its comprehensive investigation into the response of polyurea and polyurethanes to varying strain rates. Through dynamic and compressive testing, they revealed the significant impact of strain rates, particularly in conditions simulating high-velocity impacts. The study conducted by Sarva et al. [15] provided valuable insights into the stress–strain characteristics of thermoplastic polyurethane elastomers and thermoset polyurea across a wide range of strain rates, elucidating their enhanced mechanical performance as protective coatings under rapid loading conditions. The tensile properties of polyurethane were meticulously investigated by Liao et al. [16] in a separate study, encompassing a moderate range of strain rates and various temperatures. Fan et al. [17] employed the split Hopkinson pressure bar (SHPB) technique to explore the dynamic behavior of soft polymers, offering an in-depth look at the associated deformation and fracture processes. Additionally, Sassi et al. [18] conducted experimental research on the high-strain-rate compression of polyester resin matrices, revealing the material’s sensitivity to strain rate variations and formulating an empirical model that accounts for these effects. The aforementioned studies indicate that PUEs exhibit highly nonlinear stress–strain behavior and a pronounced strain rate effect at high strain levels, behaving in a rubber-like manner at low rates and transitioning to a leather-like behavior at high rates.

The constitutive relationship of PUEs plays a pivotal role in investigating their mechanical properties and is indispensable for the design and parameter optimization of energy-absorbing structures, directly impacting their applications in energy absorption and sound absorption [19,20,21]. In recent years, numerous scholars have developed constitutive equations to describe the behavior of rigid polyurethane foams and rubbery materials under both slow and rapid deformation [22,23,24,25,26]. Yang et al. [27] developed a model that characterizes the mechanical properties of elastic polyurethane foams with varying densities, with particular emphasis on their response to deformations at intermediate to high strain rates. Based on the principles of both nonlinear and linear viscoelastic theories, Wang et al. [28] developed a nonlinear viscoelastic model. The key feature of this model lies in the utilization of stress relaxation functions characterized by two distinct relaxation times. However, this model has limited applicability in capturing the dynamic responses of solid polymers under significant strains, thus failing to elucidate the complex nonlinear phenomena observed in such conditions. Yang et al. [29] proposed a viscoelastic polymer model that combines the static hyperplastic behavior of incompressible rubber materials with the viscoelastic material model, effectively capturing the mechanical response of rubber materials, particularly in scenarios involving large strain. While numerous investigators have developed constitutive models for rubber using strain energy functions to predict material behavior under slow deformation rates, there is limited research on material models under dynamic loading [30,31,32,33].

The present study involves the preparation of PUE samples at three different density levels, followed by an exploration of their mechanical response through quasi-static and dynamic SHPB tests. According to the theory of strain energy function, we develop a constitutive model that accurately characterizes the stress–strain behavior of PUE under high-velocity impact. The accuracy of the model parameters is verified through fitting analysis.

## 2. Material and Experimental Methods

### 2.1. Raw Materials and Reagents

In this study, the preparation of polyurethane (PUE) samples involved the meticulous selection of raw materials to ensure the performance and quality of the samples. Polytetramethylene ether glycol (PTMEG), serving as the main soft segment material, was procured from Wanhua Chemical Group Co., Ltd. (Yantai, China), with molecular weights of 1000 and 2000. The hard segment was derived from polyoxypropylene glycol (EP330), supplied by Shandong Bluestar Dongda Co., Ltd. (Zibo, China), with a molecular weight of 5000. 4,4′-Methylene diphenyl diisocyanate (MDI), as the starting material for the hard segment, was also provided by Wanhua Chemical Group Co., Ltd., ensuring the industrial-grade quality of the raw materials. 1,4-Butanediol (BDO) and bis (dimethylamino) ethyl ether (BDMAEE) were used as auxiliary materials, both of analytical grade, purchased from Shanghai Macklin Biochemical Technology Co., Ltd. (Shanghai, China) and Ye Innovation Materials (Shanghai, China) Co., Ltd. (Shanghai, China), respectively. Additionally, dibutyl tin dilaurate (T12), as a catalyst, was also of analytical grade and supplied by Shanghai Macklin Biochemical Technology Co., Ltd. Foaming agents AK7703 and M9956, both of analytical grade, were provided by Jiangsu Meishide Chemical Co., Ltd. (Nanjing, China), to optimize the foam structure. Lastly, the blowing agent used was homemade deionized water, ensuring its purity and consistency.

### 2.2. Preparation Process

Given water’s dual role as a blowing and chain-extending agent, raw material dehydration was essential. We then prepared Component A by blending polyether polyol PTMEG, BDO as a chain extender, organotin T12 gel catalyst, and foam regulators AK7703/M9956 (which control the closed-cell content of the foam by adjusting their quantities) with water at precise ratios. In a nitrogen-purged environment, a three-neck flask fitted with a thermometer and stirrer received the measured polyether polyol and MDI, which were heated to 85–90 °C and reacted for 4 h to form the necessary prepolymer B. Components A and B were combined, mixed thoroughly, vacuum degassed, and cast into a release agent-coated mold. The cast was cured at 70 °C for 24 h and then at 23 ± 2 °C for 7 days to yield the polyurethane flexible foam. Laser cutting was employed to fabricate test samples, ensuring precise geometric dimensions. The paper utilized specimens with densities of 0.4, 0.6, and 0.8 g/cm^3^.

### 2.3. Morphological Characterization and Particle Size Analysis

The morphology of the original samples was observed using a field emission scanning electron microscope (FESEM S4800, Switzerland Metler Toledo, Switzerland). The samples were cut into fragments and then sputter-coated with gold for SEM observation. The scanning electron microscope was operated at an acceleration voltage of 5.0 kV. The surface morphology images were analyzed using the ImageJ v1.0 analysis software to obtain the cell diameter of the foam.

### 2.4. High Strain Rate Compression Test

The focus of this study was to examine the response of PUEs under elevated strain rates, utilizing the renowned SHPB setup—a standard method for conducting such analysis. The application of the SHPB to polymers such as PUEs, however, presents certain challenges. At room temperature, PUEs exhibit a lower modulus of elasticity and significantly lower wave impedance compared to metals, resulting in attenuated signal strength when standard metal bars are employed. The obtained signal is weakened, posing challenges in securing the necessary data for a stress–strain diagram. To address this issue, various strategies can be employed to enhance signal strength, such as utilizing highly sensitive strain gauges or opting for transmission bars made of low-impedance materials like aluminum, nylon, or polymethylmethacrylate (PMMA).

**Figure 1:**
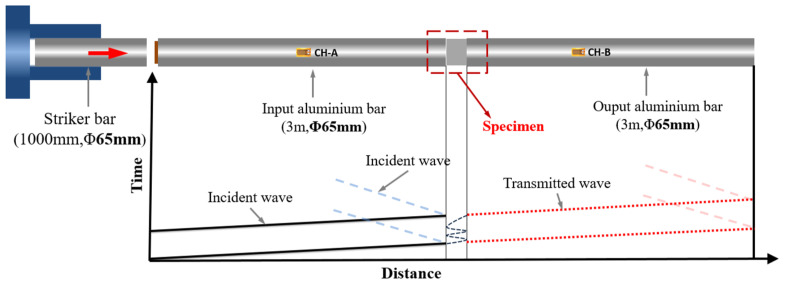
Diagram of SHPB experimental system.

The setup of the SHPB system is depicted in Figure 1, showcasing aluminum alloy bars serving as incident and transmission bars that have been engineered to minimize impedance mismatch. These bars are equipped with axial strain gauges, strategically positioned at the midpoint of the incident bars and 0.3 m away from the specimen-rod interface, enabling precise monitoring of elastic strain wave propagation. The initial installation involved strain gauges with a sensitivity factor of 2.14 on the bars. In order to enhance the detection capability for minor strain signals in this study, we opted for semiconductor strain gauges with an improved sensitivity of 110, which were then mounted onto the transmission bars.

The specimens used for quasi-static evaluation had dimensions of 40 mm in each direction, while those used for dynamic assessment with the SHPB setup were cylindrical, with a diameter of 15 mm and a thickness of 5 mm. Each density category underwent triplicate quasi-static testing as well as SHPB dynamic tests. Consistent concordance was observed among the results obtained from the replicate tests.

### 2.5. Repeatability Verification

**Figure 2:**
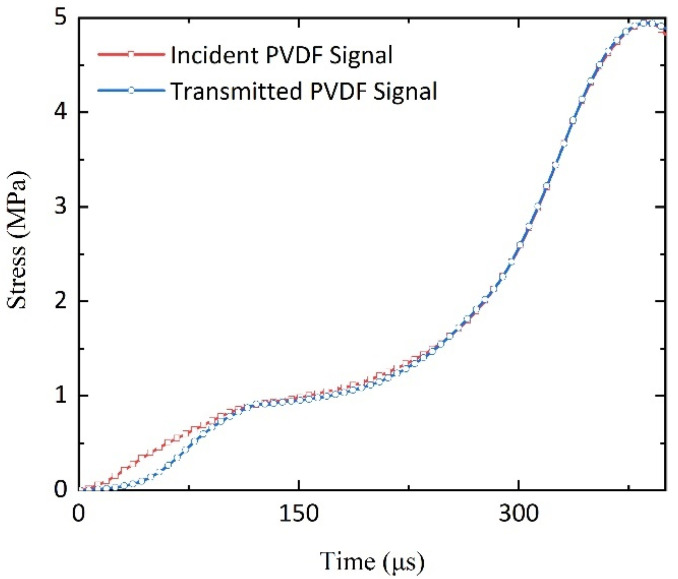
Stress–time curves at both ends of the specimen.

The maintenance of stress equilibrium throughout the test specimen is an essential prerequisite in the SHPB testing protocol during load application. Given the reduced wave impedance inherent in porous polymers, achieving stress equilibration can be a time-consuming endeavor. Within the scope of our research, stress prerequisites were met through meticulous regulation of the impact velocity and extension of the incident wave’s rise time. Furthermore, stress equilibrium was validated by directly measuring stress–time curves on the specimen surfaces using a diaphragm manometer fabricated from PVDF film, as illustrated in Figure 2.

The stress–time curve for a polyurethane elastomer under high strain rates, as depicted in Figure 2, exhibits a significant discrepancy in stress between the leading and trailing surfaces during the initial loading phase. This stage is characterized by a prominent stress gradient across the specimen. However, as the loading progresses, the distribution of stress becomes more uniform, with both front and rear surface stresses converging to equivalent levels. This convergence indicates that the specimen undergoes deformation within a state of equilibrium stress, which is considered a fundamental characteristic.

**Figure 3:**
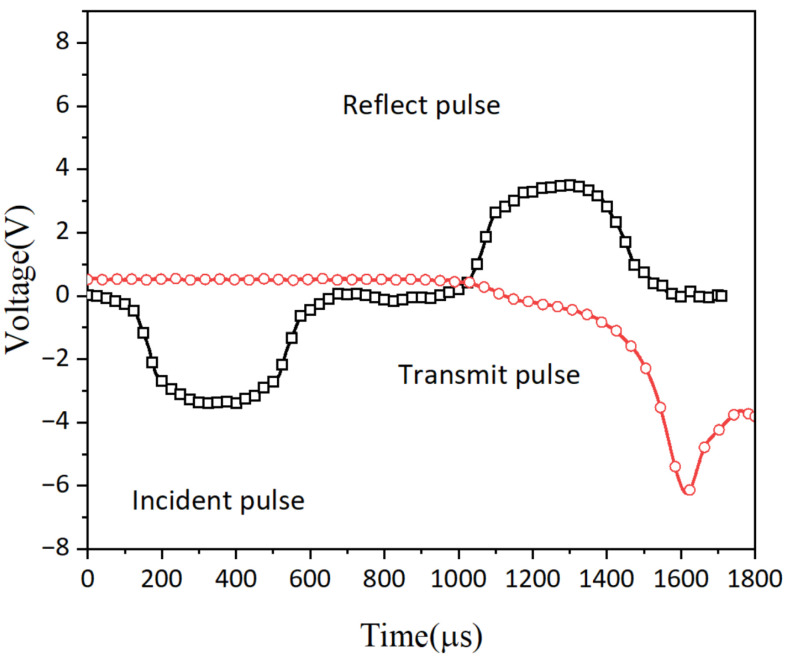
Typical stress waveform curve.

The signal curve in Figure 3 illustrates a slight but noticeable increase in the breadth of the transmitted wave compared to the incident wave. This can be attributed to the polymer foam’s inherent high compressibility and viscous properties, which allow for elongation of the stress wave during traversal. The reduced length of the reflected wave, as compared to the incident wave, is a result of overlapping reflections at both the test terminus and point of impact. This outcome is primarily achieved by intentionally reducing the span of the transmitted wave beyond the strain gauge, ensuring clear differentiation between incident and reflected waves.

**Figure 4:**
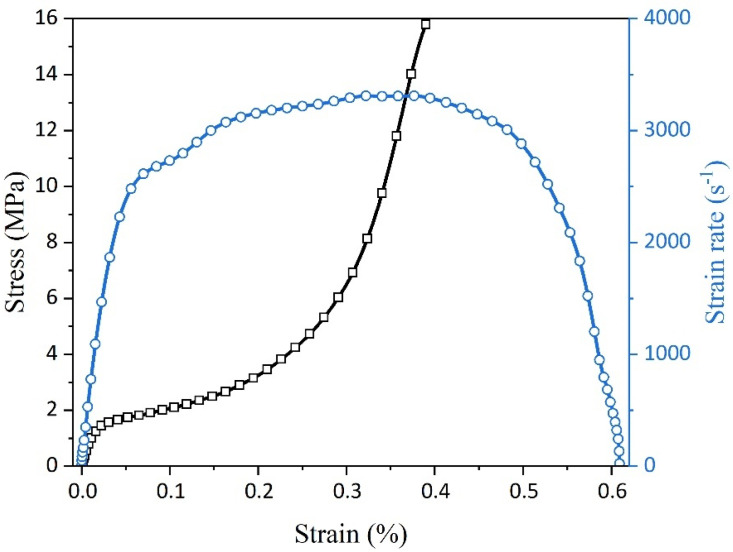
Relationship between the stress and strain rate and strain.

The fluctuation of the stress and strain rate in relation to strain is depicted in Figure 4. Upon observing the graph, it becomes evident that the strain rate rapidly escalates to a peak and subsequently oscillates around an equilibrium value until reaching the drop point, which signifies the initiation of the unloading phase. Consequently, calculations for stress and strain derived from transmitted and reflected waves were limited to the period preceding the drop point. By isolating relevant segments of stress waves, a comprehensive understanding of dynamic stress–strain behavior encompassing various compression phases was obtained across a range of strain rates.

## 3. Results and Discussion

### 3.1. Experimental Stress–Strain Results

**Figure 5:**
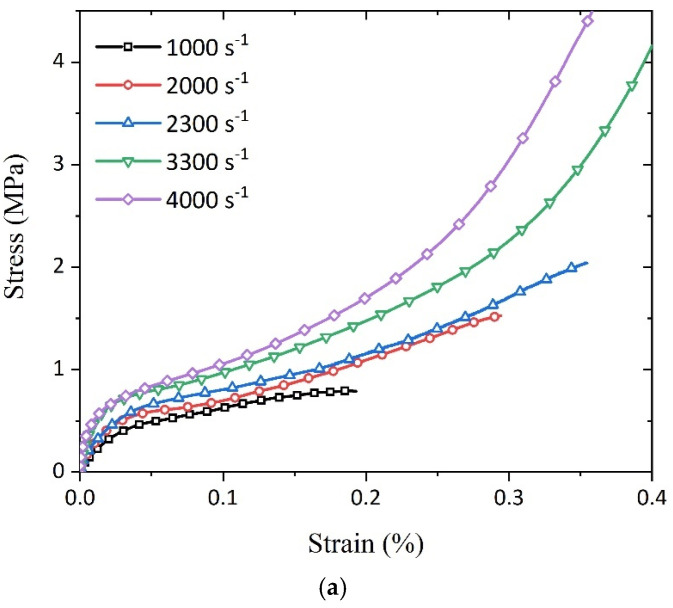

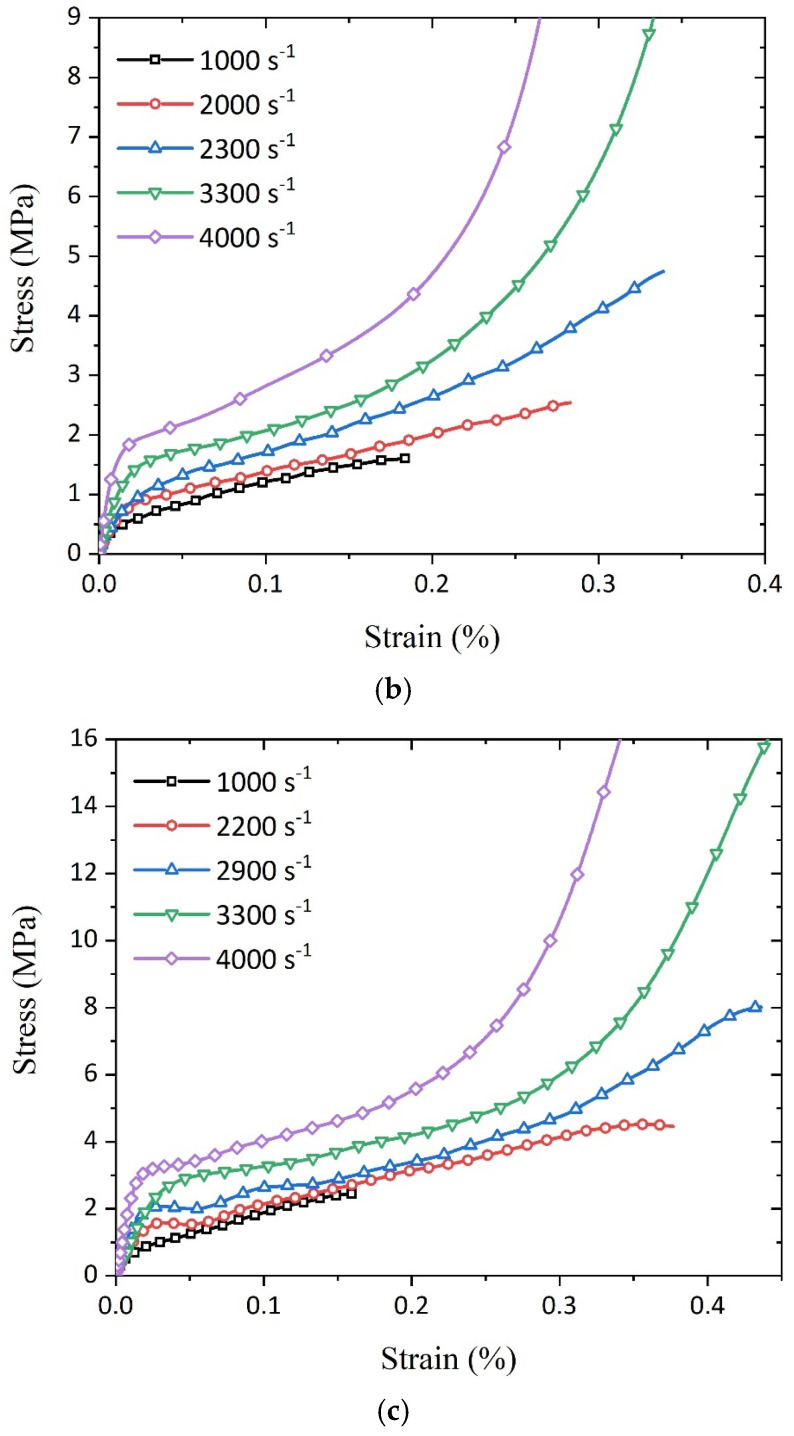
Stress–strain curves of PUEs under SHPB compression at different densities: (**a**) 0.4 g/cm^3^; (**b**) 0.6 g/cm^3^; (**c**) 0.8 g/cm^3^.

In our study, we utilized the Split Hopkinson Pressure Bar (SHPB) apparatus to conduct an in-depth analysis of the dynamic compression behavior of a polyurethane elastomer (PUE) under various strain rates. Employing the energy index method, we evaluated key mechanical parameters of PUE samples with different densities under dynamic loading conditions, including collapse stress, plateau stress, energy absorption capacity, and densification strain, as summarized in Table 1. These parameters are crucial for understanding the material’s behavior under high-speed impact. Our findings reveal that both the collapse stress and plateau stress of the PUE significantly increased with density, as shown in Figure 5a–c. Specifically, the collapse stress ranged from 0.45 MPa to 3.22 MPa, while the plateau stress increased from 0.64 MPa to 4.36 MPa, highlighting the sensitivity of mechanical properties to density fluctuations. This discovery is consistent with the results of Chen et al. [34], who observed that both the compressive strength and toughness of the PUE increased with density. Our study further uncovered the dynamic variations in this density effect under different strain rate conditions. Notably, at high strain rates, we found that the impact of density on the mechanical properties of the PUE was more pronounced, possibly related to the internal structure’s response to rapid loading.

Additionally, our data indicate that as the strain rate increases, the collapse stress and plateau stress of the PUE also increase. This trend aligns with the research by Zhang [35], who found that under high strain rates, the dynamic modulus and energy absorption capacity of the PUE were enhanced. Our study further supplements this observation by quantitatively analyzing the mechanical response differences displayed by PUEs with varying densities under different strain rates. It is worth noting that our research results differ from those of Kimura and Koyama [36] in some aspects. They reported that at low strain rates, the mechanical properties of PUEs are insensitive to density changes. These differences may be attributed to varying experimental conditions, such as loading rates, sample preparation methods, or testing environments. Our study provides a more comprehensive understanding of the dynamic compression behavior of PUEs by testing over a broader range of strain rates, thereby revealing the combined effects of density and strain rate on the mechanical properties of PUEs.

### 3.2. Effect of Strain Rate

The strain rate sensitivity is commonly quantified by the strain rate sensitivity coefficient (*m*). Specifically, the strain rate sensitivity coefficient represents the logarithmic derivative of the material flow stress in relation to the strain rate, where *m* denotes the strain rate sensitivity [37]. A typical definition for the relationship between yield stress and strain rate is as follows:

(1) $$\sigma =C{\dot{\varepsilon }}^{m}$$

(2) $$m={\left(\partial \ln\sigma /\partial \ln\dot{\varepsilon }\right)}_{T,\varepsilon }$$

**Figure 6:**
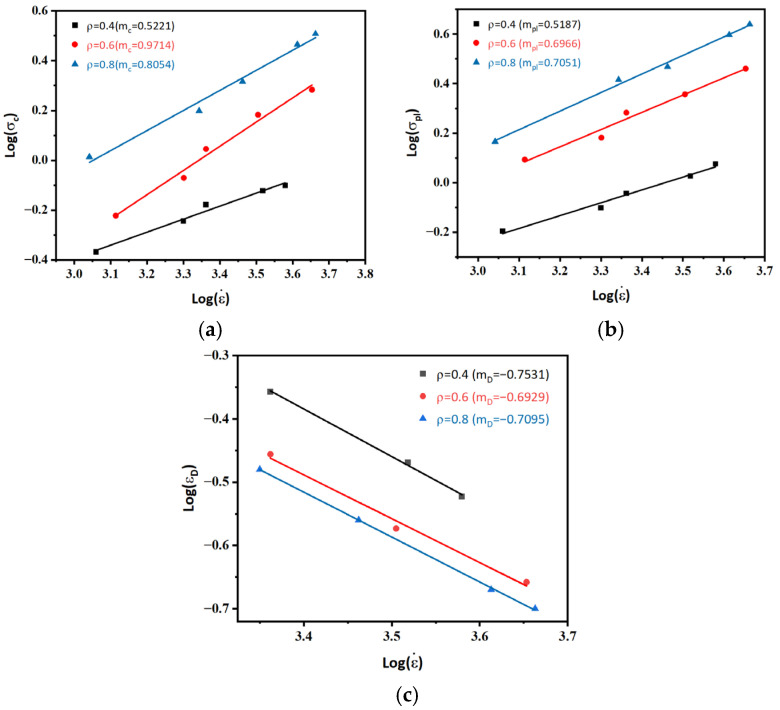
(**a**) Collapse stress, (**b**) plateau stress, and (**c**) densification strain curve with varying strain rates.

The collapse stress and plateau stress of PUEs exhibit a significant increase with the average density at different strain rates ($\dot{\varepsilon }$ = 1000–5000 s^−1^), as shown in Figure 6a–c.

Plateau stresses and densification strains were quantified using an energy-based approach. Energy absorption efficiency is given by

(3) $$\eta (\varepsilon )=\frac{1}{\sigma (\varepsilon )}\int_{\varepsilon_{y}}^{\varepsilon }\sigma (\varepsilon )d\varepsilon$$

where *ε_y_* represents the yield strain of the foam, approximately 0.2%. By definition, the densification strain *ε_D_* is the strain corresponding to the maximum value, defined as follows:

(4) $${\left.\frac{d\eta (\varepsilon )}{d(\varepsilon )}\right|}_{\varepsilon =\varepsilon_{D}}=0$$

The plateau stresses are calculated as follows:

(5) $$\sigma_{pl}=\frac{\int_{\varepsilon_{y}}^{\varepsilon }\sigma (\varepsilon )d\varepsilon }{\varepsilon_{D}-\varepsilon_{y}}$$

The experimental results show that the deformation of the foam under high-velocity impact loading affects the strain rate sensitivity of PUEs. Changes in the microstructure of the foam due to deformation significantly affect its mechanical properties. Collapse stress (*σ_c_*) and platform stress (*σ_pl_*) were plotted against strain rate for PUEs using logarithmic collocation method, as shown in Figure 6a,b.

The PUE with a higher strain rate sensitivity index, m, exhibits greater responsiveness to strain rates. Based on the study results, it can be observed that for each density, the collapse sensitivity (mc) demonstrates a stronger dependence on strain rate compared to the plateau stress (*m_pl_*). Furthermore, both the collapse sensitivity (*m_c_*) and plateau stress (*m_pl_*) increase with increasing density. The densification strain (*ε_D_*) is a crucial parameter to consider after compressing the foam pore wall. Therefore, logarithmic curves of log($\dot{\varepsilon }$) and log(*ε_D_*) were plotted to calculate the sensitivity to densification strain, as shown in Figure 6c. As the strain rate increases, there is a gradual decrease in densification strain, leading to a significantly shorter PUE platform region under high-strain-rate conditions. Additionally, it can be observed that the densification’s strain rate sensitivity (*m_D_*) remains relatively consistent across different densities.

### 3.3. Effect of Density

**Figure 7:**
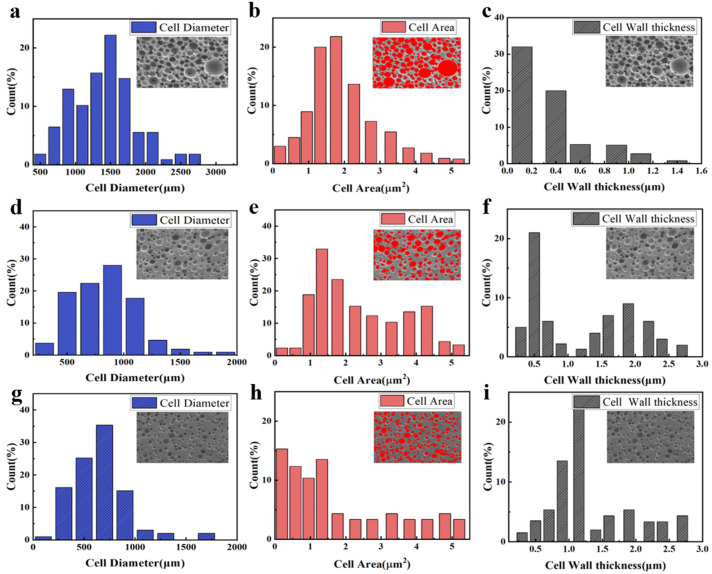
The micro-CT images were analyzed to determine the cell diameter, cell area, and cell wall thickness at different relative densities: (**a**–**c**) 0.4 g/cm^3^; (**d**–**f**) 0.6 g/cm^3^; (**g**–**i**) 0.8 g/cm^3^.

In this study, we reveal a close relationship between the microstructure of a polyurethane elastomer (PUE) and its density. The diameter, area, and cell wall thickness of PUE cells can be efficiently measured using ImageJ v1.0 software. After the high-resolution images of the PUEs were acquired by means of the SEM scanning technique and pre-processed, the cells were labeled and measured using the tools of ImageJ. The results of the quantification of microscopic parameters are shown in Figure 7. As density increases, we observed a decrease in cell volume, which is directly correlated with an increase in cell wall thickness. Specifically, with higher density, the cell walls become thicker. For instance, at a low density of 0.4 g/cm^3^, the average cell wall thickness is 0.12 µm, which increases to 1.34 µm at a high density of 0.8 g/cm^3^, representing more than a tenfold increase. This significant increase in cell wall thickness may render the material harder, stronger, and less prone to deformation.

To further substantiate our observations, we meticulously calculated the changes in cell cross-sectional area and diameter as density increased from 0.4 g/cm^3^ to 0.6 g/cm^3^. We found that the cell cross-sectional diameter decreased from 1500 µm to 800 µm, and the cell cross-sectional area reduced from 1.78 µm^2^ to 0.8 µm^2^. These changes are statistically significant and have a direct impact on the material’s macroscopic mechanical properties. The thicker cell walls provide more resistance, thereby enhancing the material’s compressive strength and resistance to deformation. Additionally, the larger cross-sectional area increases the cavity volume, which further amplifies the positive impact of enclosed air on the macroscopic mechanical properties of the material.

**Figure 8:**
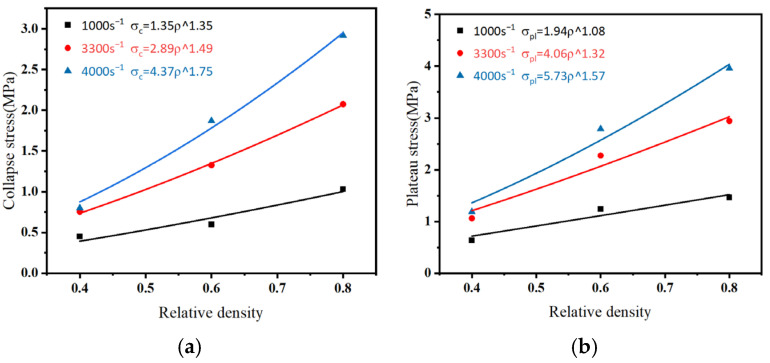

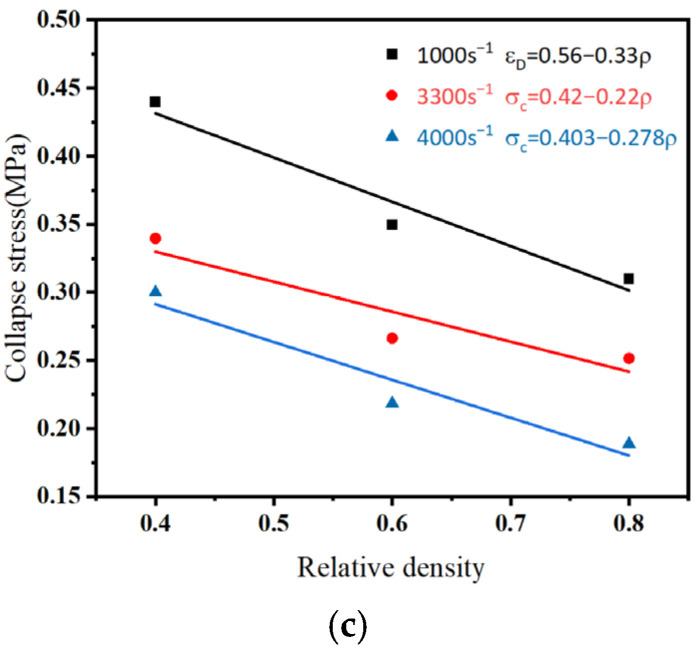
(**a**) Collapse stress, (**b**) plateau stress, and (**c**) densification strain curve with strain rate curve with varying strain rates.

The collapse and plateau stresses of the specimens increased with increasing density at different strain rates. The effect of density on collapse and plateau stresses can be described using a power relationship:

(6) $$\sigma =a\rho^{b}$$

where *a* and *b* are the parameters. The fitted parameters *a* and *b* determine the collapse stress (*σ_c_*) and plateau stress (*σ_pl_*), which exhibit a density-dependent relationship at different strain rates, as illustrated in Figure 8a,b.

The densification strain can be characterized as follows:

(7) $$\varepsilon_{D}=\beta -\alpha \cdot \rho$$

where *α* and *β* are the parameters. The inconsistency of *α* and *β* values can be observed, as they are dependent on different loading strain rates.

## 4. Constitutive Model

### 4.1. Constitutive Model Based on Strain Energy

The development of constitutive models is crucial for accurately predicting and comprehending the mechanical behavior of polymers. The aforementioned models facilitate the depiction of the intricate correlation between stress and strain under diverse loading conditions. The use of constitutive models derived from strain-energy function theory has become widely accepted in recent years for quasi-static loading conditions. Assuming that volume behavior remains incompressible, the relationship between stress and strain in a polymer material under uniaxial loading is characterized by the following formulation, as detailed in reference [31]:

(8) $$\sigma =2\left(\lambda_{2}-\frac{1}{\lambda }\right)\cdot \left(\frac{\partial U}{\partial I_{1}}+\frac{1}{\lambda }\frac{\partial U}{\partial I_{2}}\right)$$

where *λ* is the stretch ratio; *I*_1_ and *I*_2_ are strain invariants,

(9) $$I_{1}=\lambda^{2}+\frac{2}{\lambda }$$

(10) $$I_{2}=\frac{1}{\lambda^{2}}+2\lambda ;$$

and *U* is the strain energy, written as

(11) $$U=\sum_{i=0,j=0}^{\infty }C_{ij}{\left(I_{1}-3\right)}^{i}{\left(I_{2}-3\right)}^{j}$$

where the term *C_ij_* denotes a polynomial coefficient. Nonetheless, the linear model is inadequate for polyurethane elastomers due to their pronounced tendency to exhibit nonlinear characteristics under significant strains. Furthermore, experimental results indicate a highly noticeable strain-rate sensitivity in PUEs, necessitating the inclusion of the strain-rate effect in their constitutive model for an accurate description of their mechanical response.

### 4.2. Constitutive Model with Strain Rate and Density

There are several specific forms of the strain-energy function, given by Arruda–Boyce [32], Mooney–Rivlin [30], neo-Hookean [36], and Ogden [37]. Brown [38] has discussed how the number of terms in U affects the resulting stress–strain curves. Based on his results, three terms in the polynomial series are sufficient to fit the test data. The strain-energy function was given as [39]

(12) $$U=C_{1}\left(I_{1}-3\right)+C_{2}\left(I_{2}-3\right)+C_{3}{\left(I_{2}-3\right)}^{2}$$

where *C*_1_, *C*_2_, and *C*_3_ are constant polynomial coefficients. The stretch ratios are formulated accordingly for an incompressible material undergoing deformation in a radially symmetric manner.

(13) $$\lambda_{1}=\lambda ,\lambda_{2}=\lambda_{3}=\lambda^{-\frac{1}{2}}$$

where *λ* is the stretch in the loading direction, and

(14) $$\lambda =1-\varepsilon_{E}$$

where *ε_E_* denotes the engineering strain. The relationship governing the material’s behavior, as derived from its strain-energy function under uniaxial conditions, is expressed by the following equation:

(15) $$\sigma_{sef}=2(\lambda_{2}-\frac{1}{\lambda })\cdot (\frac{\partial U}{\partial I_{1}}+\frac{1}{\lambda }\frac{\partial U}{\partial I_{2}})$$

where *σ_sef_* is the true stress in the material.

By substituting Equation (12) into Equation (8), one can derive:

(16) $$\sigma_{sef}=2(\lambda_{2}-\frac{1}{\lambda })\cdot (A_{1}+A_{2}\frac{1}{\lambda }+2A_{3}\frac{1}{\lambda^{3}})$$

where *A*_1_, *A*_2_, and *A*_3_ are combinations of *C*_1_, *C*_2_ and *C*_3_.

(17) $$A_{1}=C_{1}+3C_{3}$$

(18) $$A_{2}=C_{2}-6C_{3}$$

(19) $$A_{3}=C_{3}$$

**Figure 9:**
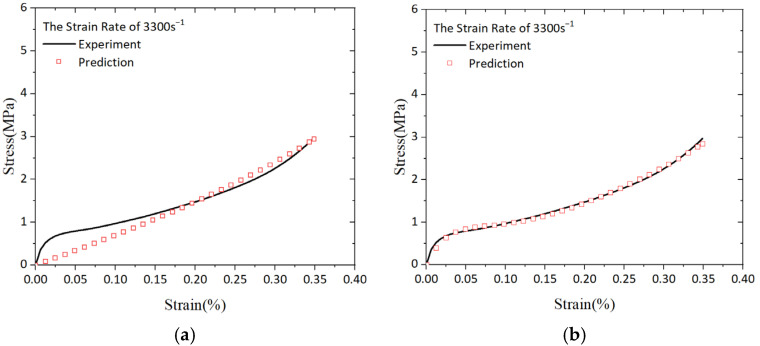
Predicted versus experimental stress–strain curves for PUE at a strain rate of 3300 s^−1^: (**a**) model by Equation (16); (**b**) model by Equation (20).

By considering $\dot{\varepsilon }$ = 3300 s^−1^ as a benchmark for the strain rate and utilizing Equation (16) based on the strain-energy function, we compared experimental results with our model’s true stress–engineering strain curves in Figure 9a. It is evident that Equation (16) effectively characterizes material behavior under high strains while also demonstrating potential for capturing nonlinearity. Nevertheless, there exists a discrepancy between our fitted equation and experimental data when examining small strains. Henceforth, it becomes crucially important to enhance our model’s ability in describing PUE’s dynamic behavior, particularly at low strains. Consequently, we developed an adjusted constitutive equation capable of accurately representing PUE’s response across both small and large strains, which was expressed as

(20) $$\sigma =\sigma_{sef}\left[1+D_{2}{\left(\frac{1}{\lambda }\right)}^{-D_{1}}\right]$$

where *D*_1_ and *D*_2_ are constant and *σ_sef_* is the true stress, which was derived from Equation (18).

The strain rate sensitivity of PUEs demonstrates a complex and nonlinear relationship with the applied strain rate, as revealed by our meticulous experimental findings. The nonlinearity is a critical aspect that significantly influences the dynamic behavior of the material. However, Equation (16), although robust, has limited applicability as it optimally describes the dynamic compression response of PUEs only under specific strain rate conditions. Therefore, it is still necessary to consider the effect of strain rate sensitivity. We developed the following model to accurately describe the dynamic characteristics of PUEs within the range of experimental strain rates, thereby enhancing the predictive capability of the model,

(21) $$\sigma =\sigma_{sef}\left[{\left(\frac{1}{\lambda }\right)}^{-B\mathrm{lg}\dot{\varepsilon }/{\dot{\varepsilon }}_{0}}+{\left(\frac{1}{\lambda }\right)}^{-D_{1}}\frac{D_{2}}{1+D_{3}\mathrm{lg}\frac{\dot{\varepsilon }}{{\dot{\varepsilon }}_{0}}}\right]$$

where $\dot{\varepsilon }$ is the strain rate; *ε*_0_ is the reference strain rate; and *B* and *D*_3_ are material constants.

Combining Equations (15) and (21), the dynamic constitutive equation of PUEs, including strain rate and large deformation effects, can be expressed as

(22) $$\sigma =2\left(\lambda^{2}-\frac{1}{\lambda }\right)\left(A_{1}+A_{2}\frac{1}{\lambda }+2A_{3}\frac{1}{\lambda^{3}}\right)\left[{\left(\frac{1}{\lambda }\right)}^{-B\mathrm{lg}\dot{\varepsilon }/{\dot{\varepsilon }}_{0}}+{\left(\frac{1}{\lambda }\right)}^{-D_{1}}\frac{D_{2}}{1+D_{3}\mathrm{lg}\frac{\dot{\varepsilon }}{{\dot{\varepsilon }}_{0}}}\right]$$

The experimental results indicate a significant density effect in PUEs, thus necessitating the inclusion of a correction term expressing this effect in the constitutive model developed for PUEs. The constitutive model was given by

(23) $$\sigma =2\left(\lambda^{2}-\frac{1}{\lambda }\right)\left(A_{1}+A_{2}\frac{1}{\lambda }+2A_{3}\frac{1}{\lambda^{3}}\right)\left[{\left(\frac{1}{\lambda }\right)}^{-B\mathrm{lg}\dot{\varepsilon }/{\dot{\varepsilon }}_{0}}+{\left(\frac{1}{\lambda }\right)}^{-D_{1}}\frac{D_{2}}{1+D_{3}\mathrm{lg}\frac{\dot{\varepsilon }}{{\dot{\varepsilon }}_{0}}}\right]\cdot \exp\left[\eta \left(1-\frac{\rho }{\rho_{0}}\right)\right]$$

where *η* represents constants; *ρ* is the initial density of the PUE; and *ρ*_0_ is a reference density for PUEs.

### 4.3. Application of Constitutive Model

**Figure 10:**
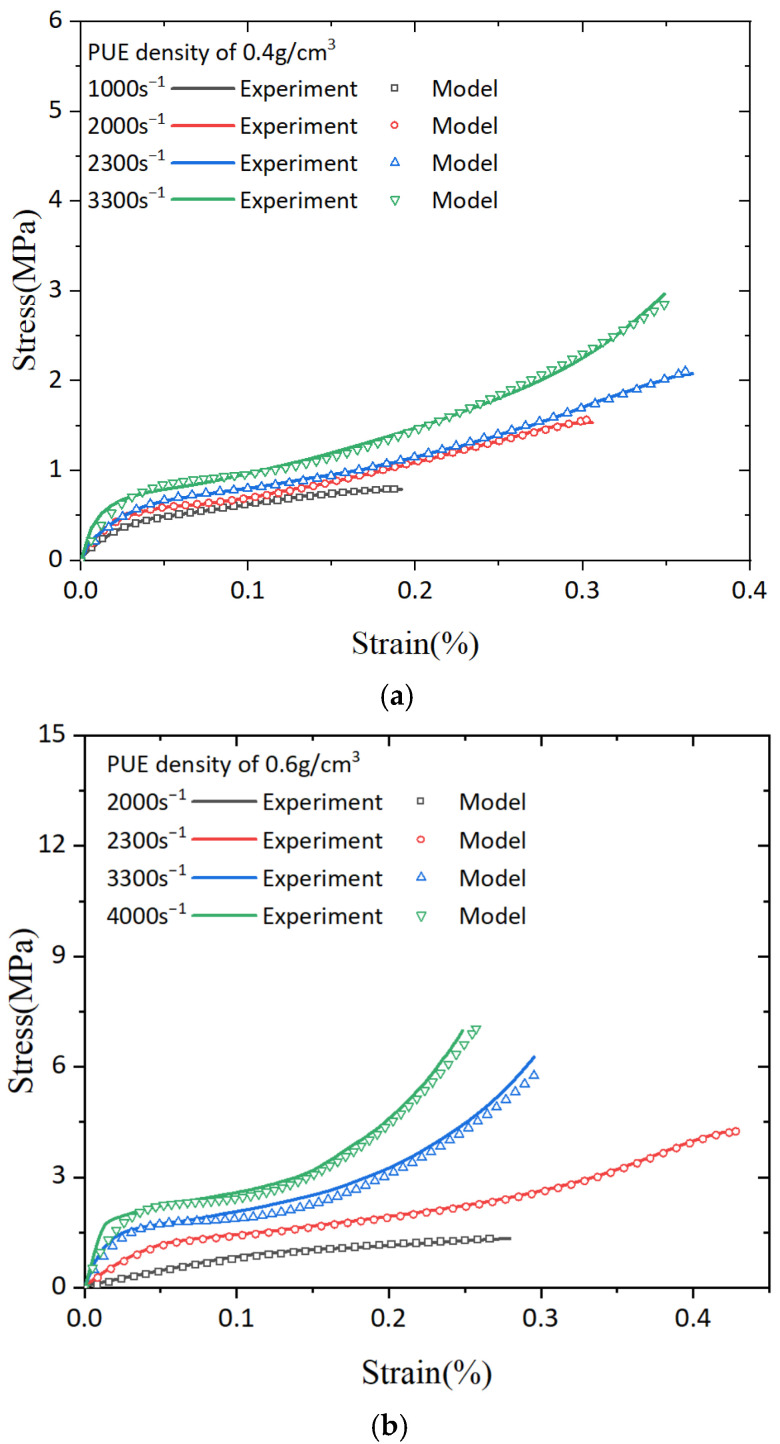

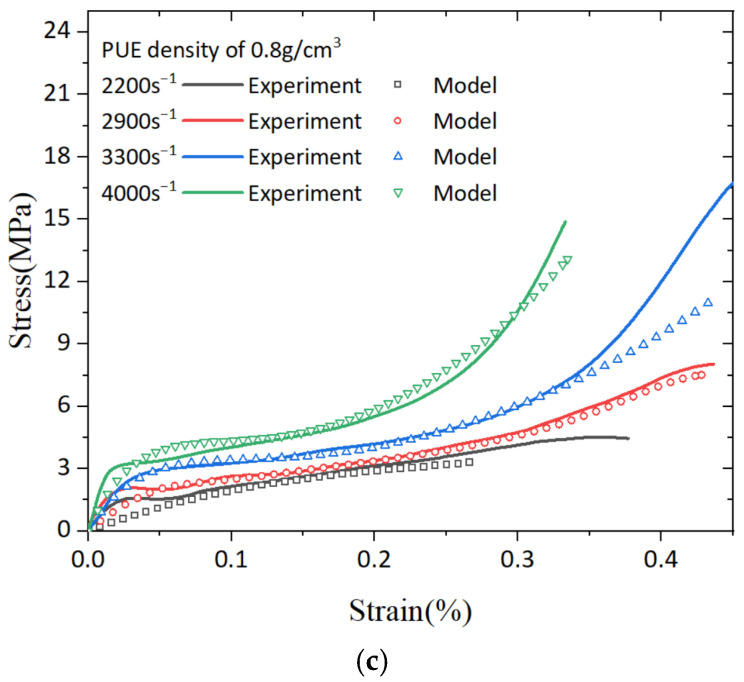
The fitting results between the experimental data and the model: (**a**) 0.4 g/cm^3^, (**b**) 0.6 g/cm^3^; and (**c**) 0.8 g/cm^3^.

The stress–strain curves obtained from dynamic tests of PUEs at varying densities are compared in Figure 10a, both as predicted by our model and as measured experimentally. The model parameters were determined through a fitting process using the least squares method, applied to Equation (23). The resulting parameters are summarized in Table 2. By comparing the experimental results of PUEs at different densities with the predicted outcomes, a strong correspondence is observed between the model’s predictions and the experimental findings depicted in Figure 10. The accuracy of our developed constitutive model in capturing the dynamic compressive response of PUEs within the examined strain and strain rate ranges is successfully validated.

## 5. Conclusions

The present study successfully fabricated PUE specimens with three different densities: 0.4 g/cm^3^, 0.6 g/cm^3^, and 0.8 g/cm^3^. A comprehensive experimental approach was employed by means of SHPB testing. The findings reveal a significant amplification in the PUE’s stress response as strain rates and densities increased, underscoring the material’s susceptibility to these variables. The strain rate sensitivity index and the density sensitivity index were employed to quantify this reactivity, confirming the material’s significant responsiveness to both parameters. The attributes of cell diameter, area, and cell wall thickness were examined through a comprehensive microstructural analysis. It was observed that an increase in density corresponded to a thickening of the cell walls, thereby potentially enhancing the material’s hardness, strength, and resistance to deformation.

The stress–strain behavior of PUEs under high-speed impacts was characterized by developing a constitutive model based on strain energy function theory. The model’s predictions closely aligned with experimental outcomes, validating its precision and reliability. The aforementioned model provides valuable theoretical insights for the implementation of PUEs in engineering, particularly in applications involving energy absorption and sandwich structures. Furthermore, it enhances our understanding of the mechanical performance of PUEs under dynamic loads, which is crucial for the strategic development and application of materials in high-demand industries such as the aerospace, automotive, and construction industries.

## Figures and Tables

**Table 1:** Mechanical properties of PUEs obtained by SHPB experiments.

Relative Density (g/cm^3^)	Strain Rate (s^−1^)	Collapse Stress (MPa)	Plateau Stress (MPa)	Densification Strain	Energy Absorption (MJ/m^3^)
0.4	1000	0.45	0.64	—	0.11
	2000	0.57	0.79	—	0.27
	2300	0.66	0.90	0.44	0.40
	3300	0.75	1.06	0.34	0.49
	4000	0.79	1.19	0.30	0.52
0.6	1000	0.60	1.24	—	0.20
	2000	0.85	1.52	—	0.46
	2300	1.11	1.92	0.35	0.53
	3300	1.52	2.27	0.27	0.69
	4000	1.92	2.89	0.22	0.76
0.8	1000	1.03	1.47	—	0.26
	2200	1.58	2.60	—	1.12
	2900	2.07	2.94	0.31	1.51
	3300	2.92	3.96	0.25	1.70
	4000	3.22	4.37	0.19	1.70

**Table 2:** Material constants for the constitutive model described by Equation (23).

**A_1_ (MPa)**	**A_2_ (MPa)**	**A_3_ (MPa)**	**B**	**D_1_**
−6.217	−2.233	−4.330	3.003	−0.840
**D_2_**	**D_3_**	**ε_0_ (s** **^−1^)**	***ρ*_0_ (g/m^3^)**	**η**
−0.831	−1.006	1000	0.4	−17.38

## Data Availability

The original contributions presented in the study are included in the article; further inquiries can be directed to the corresponding author.
